# Prospective Evaluation of Large Language Model Integration Into a Classical Hematology Case Conference

**DOI:** 10.2196/89939

**Published:** 2026-03-18

**Authors:** Tariq Kewan, Alfred I Lee, Layla Van Doren

**Affiliations:** 1 Department of Medicine, Division of Hematology, Mayo Clinic, 200 1st St SW, Rochester, MN, 55905, United States, 1 6193896524; 2 Department of Medicine, Division of Hematology, Yale University, New Haven, CT, United States

**Keywords:** artificial intelligence, education, machine learning, large language model, classical hematology

## Abstract

Prospective integration of large language model tools into a classical hematology challenging-cases conference was feasible, increased clinician familiarity and interest, and was perceived as diagnostically and educationally valuable.

## Introduction

Artificial intelligence (AI) systems based on large language models (LLMs) are increasingly accessible to clinicians and trainees, yet their practical use in real-time case-based learning has not been systematically evaluated [1-3]. Classical hematology, with its diagnostic challenges and frequent reliance on critical thinking represents a relevant environment for early implementation. We conducted a prospective study to assess the integration of LLM tools into a classical hematology case conference and to evaluate user experience, perceived value, and key considerations for safe adoption.

## Methods

**Figure 1.**
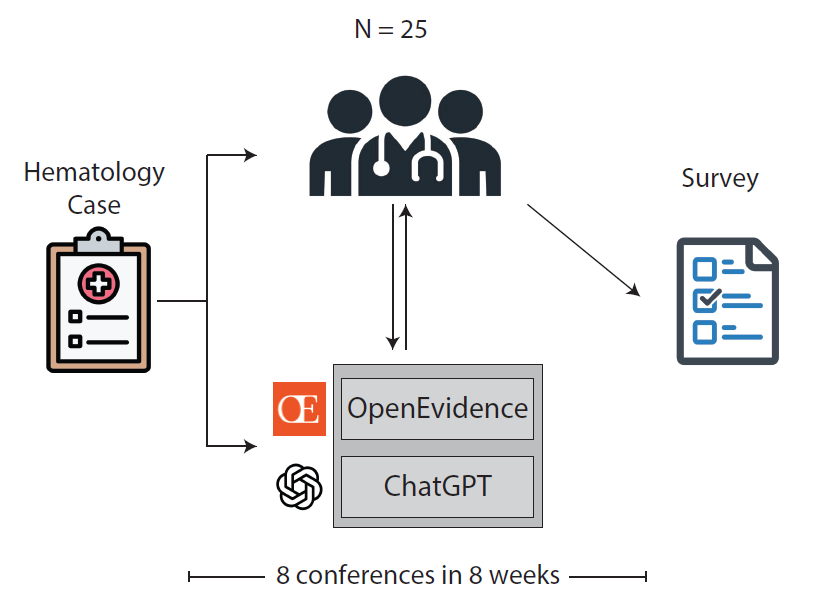
General scheme of large language models (LLMs) integration within the Classical Hematology Case Conference.

Over eight consecutive sessions, two LLMs, ChatGPT and Open Evidence AI, were incorporated into the Yale Classical Hematology Case Conference (Figure 1). The use of two distinct LLM platforms was intentional; Open Evidence AI was selected because it provides guaranteed, source-linked medical references, while the inclusion of ChatGPT aimed to demonstrate that different LLM platforms can be leveraged to support case-based discussions in classical hematology. Importantly, no formal performance comparison between the two platforms was conducted in this initiative. Presenters prepared structured prompts summarizing clinical presentation, laboratory data, and specific questions relevant to differential diagnosis and management for each case. These prompts were used to generate outputs that included differential diagnoses, diagnostic algorithms, rationale for additional workup, evidence-based therapeutic recommendations, and citation-supported references.

The AI-generated content was shown during case discussions and evaluated in parallel with expert clinical reasoning. This created a structured format in which faculty and trainees could critically evaluate LLM output, compare it with established approaches or recommendations, and examine areas of concordance and discordance.

## Methods

### Ethical Considerations

This prospective educational feasibility study involved an anonymous survey of conference participants without collection of identifiable private information. Participation was voluntary, and all data were analyzed and reported in aggregate to ensure confidentiality.

## Results

Following the intervention, 25 attendees completed a structured questionnaire (Multimedia Appendix 1). Respondents were primarily faculty hematologists (n=16/25, 64%) and trainees (n=7, 28%), with a wide range of practice experience. Prior to the intervention, only 16% (n=4) reported being “very familiar” with AI in clinical hematology; after the intervention, 36% (n=9) reported “a lot of familiarity,” and none reported no familiarity. Similarly, the proportion using AI frequently or occasionally increased from 44% (n=11) preintervention to 68% (n=afterward. These findings suggest that even limited, structured exposure can influence clinician comfort with AI tools.

Participants generally perceived AI as valuable or somewhat valuable in the context of case discussion (n=21, 84%). The aspects rated highest were the generation of alternative diagnoses (80%) and the retrieval of relevant references (92%). These findings align with known capabilities of LLMs to rapidly provide relevant information, broaden diagnostic considerations, and provide literature support that may otherwise be time-consuming to compile during real-time discussions [4,5]. In classical hematology, where cases often involve complex presentations with broad differential diagnoses, these contributions offer notable educational benefits.

Participants also identified several limitations. The most frequently reported concern was that the quality and specificity of AI output depended significantly on the structure and clarity of the input (n=15, 60%). This finding underscores the need for standardized prompting frameworks, a known issue across clinical AI applications. Respondents also noted that AI-generated treatment suggestions were sometimes insufficiently tailored to the individual clinical scenario (52%) and that occasional incomplete or irrelevant outputs were generated for both diagnoses and management options (52%). These observations reinforce the importance of clinician oversight and illustrate the current inability of LLMs to independently interpret patient-specific detailed clinical scenarios [6].

Importantly, nearly all respondents (n=23, 92%) believed that AI should function exclusively in an adjunctive capacity, supporting rather than replacing clinician judgment. Only one participant indicated that AI added little or no value to the case discussions. These perspectives mirror broader concerns in the medical community regarding reliability, safety, and the need for human supervision in clinical applications of AI [6,7].

## Discussion

Our findings demonstrate that prospective integration of LLM tools into a classical hematology case conference is both feasible and acceptable to clinicians. The experience increased familiarity with AI systems, encouraged early adoption, and was perceived as valuable in enhancing diagnostic evaluation and reference retrieval. Importantly, the intervention created a structured environment for examining limitations of AI, including prompt dependency, generalization, and incomplete reasoning pathways, thus reinforcing the need for careful oversight and transparency.

This early experience suggests several considerations for future implementations. First, structured prompt templates may improve output reliability and consistency. Second, AI integration may be best positioned as an on-demand component rather than a continuous or self-supervised feature of case presentations. Third, further prospective studies should evaluate diagnostic accuracy, effects on clinical decision-making, potential biases, and implications for trainee education. Finally, developing practical guidelines for human-AI integration is essential as educational and clinical environments adopt these tools [8,9].

In summary, our prospective feasibility study provides early evidence that integrating LLM-based tools into clinical case conferences enhances educational value and increases clinician familiarity with AI. These findings support continued, supervised exploration of AI-assisted case-based learning within hematology and other medical specialties.

## Supplementary material

10.2196/89939 Multimedia Appendix 1 Structured questionnaire .

## Abbreviations

AI: artificial intelligence
LLM: large language model

## References

[1] Lucas HC, Upperman JS, Robinson JR (2024). A systematic review of large language models and their implications in medical education. Med Educ.

[2] Sallam M (2023). ChatGPT utility in healthcare education, research, and practice: systematic review on the promising perspectives and valid concerns. Healthcare (Basel).

[3] Thirunavukarasu AJ, Ting DSJ, Elangovan K, Gutierrez L, Tan TF, Ting DSW (2023). Large language models in medicine. Nat Med.

[4] Qiu P, Wu C, Liu S (2025). Quantifying the reasoning abilities of LLMs on clinical cases. Nat Commun.

[5] Rebitschek FG, Carella A, Kohlrausch-Pazin S, Zitzmann M, Steckelberg A, Wilhelm C (2025). Evaluating evidence-based health information from generative AI using a cross-sectional study with laypeople seeking screening information. NPJ Digit Med.

[6] Asgari E, Montaña-Brown N, Dubois M (2025). A framework to assess clinical safety and hallucination rates of LLMs for medical text summarisation. NPJ Digit Med.

[7] Al-Nusair J, Lanino L, Durmaz A, Porta MGD, Zeidan AM, Kewan T (2025). Artificial intelligence in myeloid malignancies: clinical applications of machine learning in myelodysplastic syndromes and acute myeloid leukemia. Blood Rev.

[8] Ong JCL, Chang SYH, William W (2024). Ethical and regulatory challenges of large language models in medicine. Lancet Digit Health.

[9] Weidener L, Fischer M (2023). Teaching AI ethics in medical education: a scoping review of current literature and practices. Perspect Med Educ.

