# Supplementary material for: Prospective Evaluation of Large Language Model Integration Into a Classical Hematology Case Conference
Source: JMIR Form Res. 2026 Mar 18;10:e89939. doi: 10.2196/89939 (PMC12998601; doi:10.2196/89939)

Prospective Evaluation of Large Language Model Integration into a Classical Hematology Case Conference

1. What is your current professional status?

Trainee (resident or fellow)	7
Hematology attending	16
Transfusion medicine provider	0
Pharmacist	0
Advanced practice provider (PA or NP)	1
RN	1
Other	0

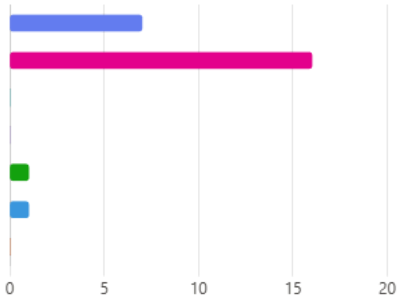

2. Years in hematology practice:

Currently in Training (fellows or residents)	7
<5	6
5-10	5
11-20	6
>20	1

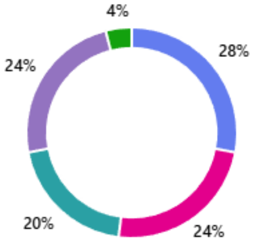

3. Prior to the use of AI in the classical hematology conference, how familiar were you with the use of AI in clinical hematology?

Very familiar	4
Somewhat familiar	12
Not familiar	9

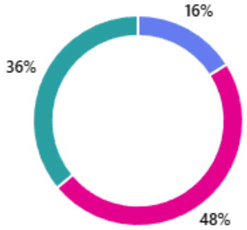

4. How often did you use AI tools in your clinical practice?

Frequently	4
Occasionaly	7
Rarely	10
Never	4

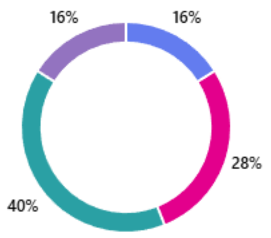

5. Which of the following best described your level of interest in incorporating AI into your clinical practice?

I had some interest in incorporating AI into my clinical practice	12
I had no interest in incorporating AI into my clinical practice	2
I was very interested in incorporating or expanding use of AI in my clinical practice	10
Other	1

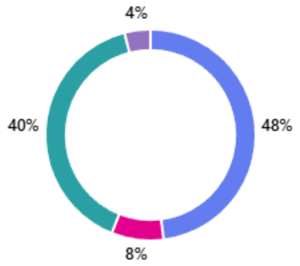

6. How familiar were you in evaluating the broader implications of AI in clinical care (e.g., legal/ethical considerations, accuracy of AI outpu t)?

Some familiarity (e.g., able to recognize legal/ethical grey zones and gauge accuracy of AI output)	14
Little to no familiarity	10
A lot of familiarity (e.g., contributed to design of studies to evaluate legal/ethical issues and accuracy...	0
Other	1

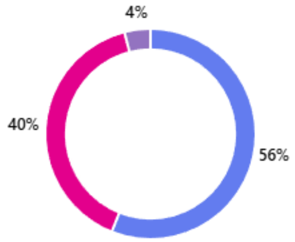

7. Which best described your experience developing AI innovations in healthcare?

I had not participated in AI model development	21
I collaborated with technical teams to apply or refine AI models for clinical use	2
I led or co-led AI development efforts, including model design, validation, and interdisciplinary...	1
Other	1

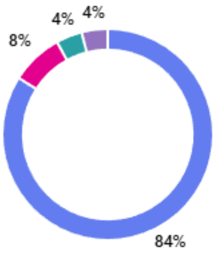

8. Now that you've seen AI in use in the classical hematology conference, how much familiarity would you say that you have with using AI in clinical hematology?

A lot of familiarity	9
Some familiarity	15
No familiarity	0

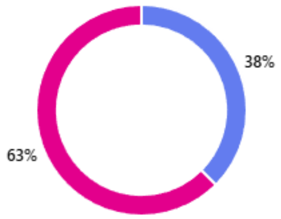

9. How often do you **currently** use AI tools in your clinical practice (e.g., clinical decision making)?

Frequently	7
Occasionaly	10
Rarely	5
Never	2

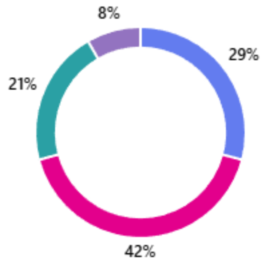

10. Which of the following best describes your level of interest in incorporating AI into your clinical practice currently?

I have some interest in incorporating AI into my clinical practice currently	10
I have no interest in incorporating AI into my clinical practice currently	0
I am very interested in incorporating or expanding use of AI in my clinical practice currently	14

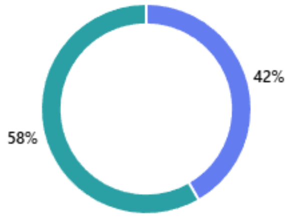

11. How familiar are you in evaluating the broader implications of AI in clinical care (e.g., legal/ethical considerations, accuracy of AI outpu t)?

Some familiarity (e.g., able to recognize legal/ethical grey zones and gauge accuracy of AI output)	16
Little to no familiarity	6
A lot of familiarity (e.g., contributed to design of studies to evaluate legal/ethical issues and accuracy...	2

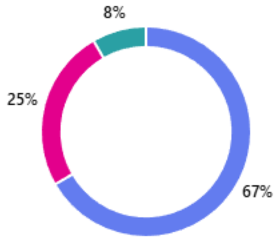

12. Which best describes your experience developing AI innovations in healthcare?

I have not participated in AI model development	19
I collaborate with technical teams to apply or refine AI models for clinical use	4
I lead or co-lead AI development efforts, including model design, validation, and interdisciplinary...	1

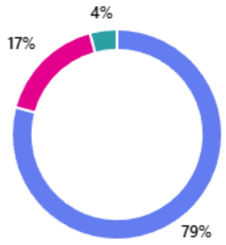

13. The AI tool contributed meaningfully to diagnostic decision-making during the conference

Agree	8
Somewhat agree	11
Neutral	5
Somewhat disagree	1
Disagree	0

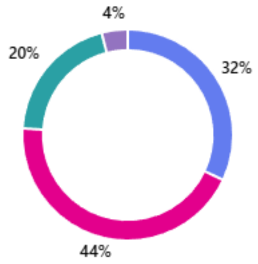

14. The AI tool contributed meaningfully to the treatment plan/management during the conference

Agree	7
Somewhat agree	9
Neutral	9
Somewhat disagree	0
Disagree	0

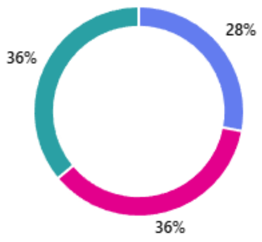

15. The AI tool provided valuable references or evidence that guided decision making.

Agree	8
Somewhat agree	8
Neutral	6
Somewhat disagree	2
Disagree	1

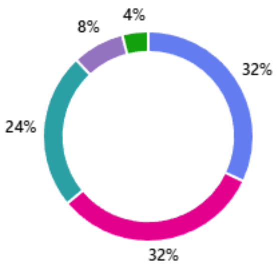

16. Overall, how would you rate the added value of AI in classical hematology case conference discussions?

Very valuable	6
Somewhat valuable	15
Neutral	1
Limited value	3
Not at all valuable	0

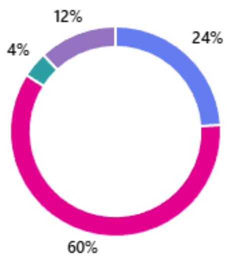

17. What ways did AI add value, if any? *(Select all that apply)*

Offering guideline-based recommendations	14
Generating evidence-based treatment options	14
Suggesting alternative diagnoses	20
Priotirzing next diagnostic steps	9
AI added little or no value	2
Other	1

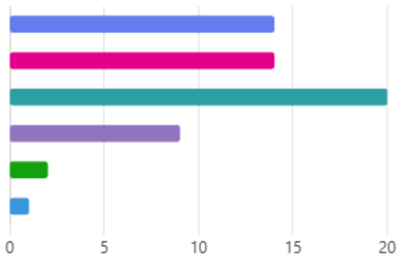

18. What limitations did you observe with the AI tool? *(Select all that apply)*

Over-reliance on existing data	5
Incomplete or irrelevant suggestions	13
Incomplete or irrelevant diagnosis	6
Incomplete or irrelevant treatment options	7
Recommendations are too general, not personalized	13
Quality of AI output was very dependent on effective wording of medical input	15
AI output was sometimes confusing	1
There were no limitations	2
Other	4

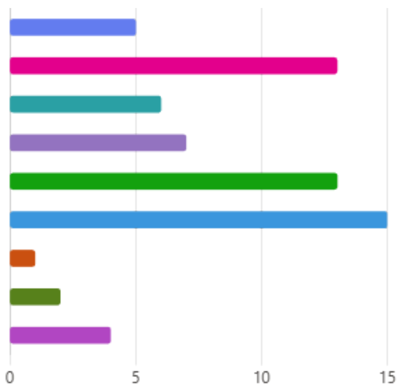

19. I would support continued or expanded use of AI in future case conferences.

Agree	16
Somewhat agree	6
Neutral	3
Somewhat disagree	0
Disagree	0

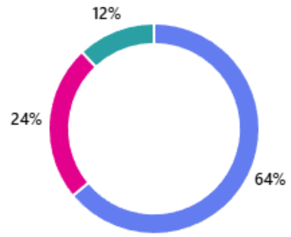

20. In which areas, if any, should AI be implemented in the classical hematology conference *(Select all that apply):*

Provide differential diagnosis	20
Assist with treatment decision making	9
Provide relevant references	23
Predicting outcomes or risk stratification	11
AI should not be used or implemented	1
Other	0

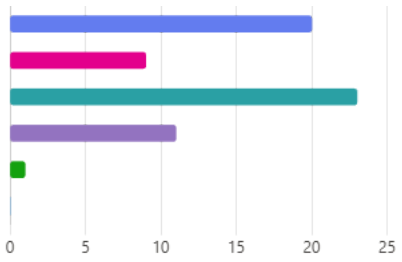

21. How should AI be incorporated into classical hematology conference (*Select all that apply)*:

As a background support tool for presenters	13
Displayed side by side during case presentation	6
As interactive tool just for specific questions	18
Used only for post presentation summaries and insights	9
Other	0

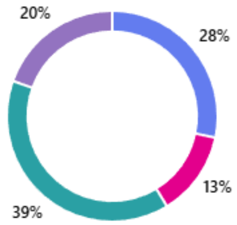

22. What are your thoughts on the use of AI in clinical decision-making

AI can function independently from providers in clinical decision making	0
AI has an adjunctive role in helping providers with clinical decision making	23
AI should not be used in clinical decision making	1
Other	1

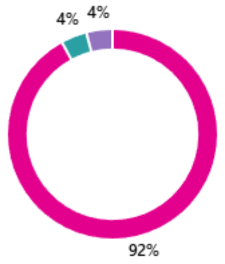

Supplement: Multimedia Appendix 1 [file formative-v10-e89939-s001.pdf]
